# Living with mentally ill parents during adolescence: a risk factor for future welfare dependence? A longitudinal, population-based study

**DOI:** 10.1186/s12889-015-1734-1

**Published:** 2015-04-22

**Authors:** Lisbeth Homlong, Elin Olaug Rosvold, Åse Sagatun, Tore Wentzel-Larsen, Ole Rikard Haavet

**Affiliations:** Department of General Practice, Institute of Health and Society, University of Oslo, PB 1130, Blindern, 0318 Oslo Norway; Centre for Child and Adolescent Mental Health, Eastern and Southern Norway, Oslo, Norway; Norwegian Centre for Violence and Traumatic Stress Studies, Oslo, Norway

**Keywords:** Mental health, Mental health problem, Depression, Family stress, Parents, Adolescence, Social support, Work marginalization, Welfare dependence

## Abstract

**Background:**

Living with parents suffering from mental illness can influence adolescents’ health and well-being, and adverse effects may persist into adulthood. The aim of this study was to investigate the relationship between parents’ mental health problems reported by their 15–16-year-old adolescents, the potential protective effect of social support and long-term dependence on public welfare assistance in young adulthood.

**Methods:**

The study linked data from a youth health survey conducted during 1999–2004 among approximately 14 000 15–16-year-olds to data from high-quality, compulsory Norwegian registries that followed each participant through February 2010. Cox regression was used to compute hazard ratios for long-term welfare dependence in young adulthood based on several risk factors in 15–16-year-olds, including their parents’ mental health problems.

**Results:**

Of the total study population, 10% (1397) reported having parents who suffered from some level of mental health problems during the 12 months prior to the baseline survey; 3% (420) reported that their parents had frequent mental health problems. Adolescent report of their parents’ mental health problems was associated with the adolescents’ long-term welfare dependence during follow-up, with hazard ratios (HRs) of 1.49 (CI 1.29–1.71), 1.82 (1.44–2.31) and 2.13 (CI 1.59–2.85) for some trouble, moderate trouble and frequent trouble, respectively, compared with report of no trouble with mental health problems. The associations remained significant after adjusting for socio-demographic factors, although additionally correcting for the adolescents’ own health status accounted for most of the effect. Perceived support from family, friends, classmates and teachers was analysed separately and each was associated with a lower risk of later welfare dependence. Family and classmate support remained a protective factor for welfare dependence after correcting for all study covariates (HR 0.84, CI 0.78–0.90 and 0.80, 0.75–0.85). We did not find evidence supporting a hypothesized buffering effect of social support.

**Conclusions:**

Exposure to a parent’s mental health problem during adolescence may represent a risk for future welfare dependence in young adulthood. Perceived social support, from family and classmates in particular, may be a protective factor against future long-term welfare dependence.

## Background

Depression and anxiety are prevalent in the adult population [1,2] and consequently a substantial number of children have parents who suffer from mental health problems. Living with parents who have mental health problems, can negatively impact psychological development and adjustment during childhood and adolescence [3-7]. As well as a genetic predisposition for mental illness in their offspring [8,9], parents’ mental health problems may affect the home environment and thus their children’s psychosocial functioning and risk of developing mental illness [10-12]. Parents may develop an unhealthy parenting style, when suffering from a mental illness, which can lead to unsecure attachments and affect cognitive and affective development in their children [3,7,13]. A contextual model explaining adverse effect on children includes socio-economic factors and adverse life experiences, including living in a stressful home environment, which can explain both mental illness in parents and maladjustments during childhood [3,7,13]. Mental health problems in parents are frequently associated with a wide range of other adversities, including family conflicts, domestic violence, divorce and poverty [13]. Thus, the adverse effects on the child may be a consequence of a clustering of several negative factors.

Early determinants of health during gestation, childhood and adolescence influence health and well-being throughout the life-span. The study of long-term effects on health and disease risk of such earlier determinants, is termed life-course epidemiology [14]. Sawyer and collegues introduced a theoretical framework on how a life course perspective unites with important social determinants, on how to understand the fundaments of adolescent health and development, a framework which is the basis of our work [15]. According to Sawyer, adolescence is a key developmental stage during the life-course, were adolescent health and well-being are affected by early life adversities, and where also adolescent circumstances influence future health and adjustment [15].

Previous studies on work marginalization have focused mainly on adult predictors. Fewer studies focus on determinants in childhood and adolescence. A Finnish longitudinal study focusing on early life adverse experiences, found a 3.5-fold risk of disability pension when the study subjects had experienced 5–6 negative events [16]. Some studies have established strong associations between socioeconomic position in childhood and health and labour market attachment in adulthood [17,18]. Health problems in childhood and adolescence are found to be assoiciated with subsequent work disability, including chronic disease during childhood, low birth weight and low gestational age [19-21]. Increasing prevalence rates of mental health problems in the young may also play a role [18,19,22,23]. Mental health problems in general [24,25], and depressive symptomatology in particular, influence adolescents’ ability to graduate from high school [26,27], and high school graduation is essential for integration into the work-force in adulthood [19,28,29]. Furthermore, diagnoses within mental health illness were the main reason for young adults receiving long-term health-related public welfare in Norway in 2008; anxiety and depression were the most common diagnoses [30]. Studies have indicated a strong relationship between parental depression and child negative mental health outcomes [31-33] and that such adverse effects persist over time [10,34]. Although increasing evidence supports associations between adolescents’ own mental health and later work marginalization, few studies have explored the relationships between their parents’ mental health and similar functional outcomes. One recent Norwegian study found associations between parent anxiety and depression and medical welfare dependence in young adulthood among their offspring [34]. However, the potential positive influence of social support was not explored in that study.

The importance of social support in adolescence for mental health, general well-being and coping is well established [35]. Social support is a complex and multidimensional construct that can be conceptualized and measured in different ways [36]. Perceived social support, i.e., an individual’s appraisal of the availability and/or adequacy of support, is perhaps the most frequently studied dimension, and has been found to have the strongest relationship with stress reduction and improved well-being [36,37]. Several studies have investigated the independent effect of perceived social support irrespective of exposure to stressors, as well as the buffer effect, which emphasizes that social support is especially important when an individual is exposed to life stress [37-39]. A previous Norwegian study showed that social support has a positive effect on adolescents’ mental health by buffering their risk of developing mental disorders when exposed to negative life events, although this effect was only significant for depression [40]. In a longitudinal study, Ystgaard and colleagues found that social support had a buffer effect against mental health problems in boys exposed to life adversities [41]. A recent Norwegian study by Stroem and colleagues found a protective effect of family and classmate support on future welfare dependence in individuals exposed to abuse and bullying during adolescence [42]. Support from family – and primarily the feeling of attachment, acceptance and trust – is considered of major importance for healthy development throughout childhood and adolescence.

Our study aimed to investigate potential long-term consequences of living with the burden of parents with mental health problems during the formative years of adolescence. This was accomplished by studying the associations between the parents’ mental health problems based on 15–16-year-old participants’ reports, and these adolescents’ subsequent welfare dependence during young adulthood. We also aimed to explore the potential protective effect of different social support dimensions in relation to welfare dependence. Assuming that parents’ mental health problems have a negative impact on their adolescents’ health and adjustment, we hypothesized that a high level of social support could have a buffering effect on dependence of welfare benefits in young adulthood.

## Methods

### Population

Baseline data were collected from a comprehensive health survey of all 10th grade secondary school students (ages 15–16) living in six Norwegian counties during 1999–2004. The youth survey was initiated in Oslo in 1999/2000 and was subsequently extended to include five more counties in the following years. A total of 18 425 10th graders were invited to participate at baseline and the overall response rate was 87% (n = 15 966). A more detailed description of how the survey was conducted has been reported previously [43]. The survey included items about relationships with family, friends and school; physical and mental health; health behaviour; and life events [44]. Detailed information about the health survey is available from the Norwegian Institute of Public Health [45].

We linked these survey data to data from Statistics Norway and the National Insurance Services (NIS/FD-trygd), compulsory national databases that supply detailed information on the entire cohort through February 2010. We had the opportunity to link the records through use of the national identification number assigned to every resident of Norway. After linkage of the data, performed by Statistics Norway, the national identification numbers were removed and the data were kept in a secure computer system; thus, confidentiality was ensured. At baseline, adolescents were asked to consent to our linking the data between the survey and national registers at a later date; 88% of the participants agreed (n = 14 062).

The NIS registry provided information on each participant’s use of welfare benefits. Residents of Norway are all insured by the NIS and employees can receive sickness benefits for up to one year if they suffer from a medical condition. Until March 2010 adults with chronic medical conditions could receive medical or vocational rehabilitation benefits with an aim to restore working ability, or they could be granted a temporary disability benefit. These three benefits were later collapsed into one single benefit called AAP (work assessment allowance). Norwegians insured by the NIS can be granted a permanent disability benefit if their condition is sufficiently severe, permanent and reduces their working capacity by 50% or more. If you are registered as a job seeker and have earned rights through former employment, you can receive unemployment benefits up to 104 weeks. In addition, a resident of Norway who is unable to care for himself or herself or for any dependents may receive a basic social security benefit irrespective of medical history. In our study, registration for welfare dependence started the calendar year when each adolescent reached 18 years of age, and lasted until end of follow-up through February 2010. As the original survey was conducted through five consecutive years, the length of the follow-up period varies between the counties. Statistics Norway also provided information on cases of death and emigration during follow-up; these cases were censored in the time to event/Cox proportional regression analyses.

### Variables

#### Main outcome

Time to receipt of the first occuring event of a long-term welfare benefit was the main outcome variable. The study subjects may have received several periods of long-term welfare benefits, but only the first event, according to the below defined criterias, is used in the analyses. We collected NIS information about each participant’s use of different welfare benefits during the follow-up period, including sickness benefits, medical and vocational rehabilitation benefits, social security benefits, unemployment benefits, temporary disability benefits and permanent disability benefit. We defined long-term receipt as either a 100% sickness benefit received at least 180 days in one year, receipt of medical or vocational rehabilitation, temporary or permanent disability pension, unemployment benefit lasting at least 180 consecutive days in one year, or use of social security support for at least six months during one year. We chose to exclude individuals who received permanent disability benefits before the age of 20 (n = 24), because a majority of those were diagnosed with intellectual disabilities, diagnoses within the autistic spectrum, or severe psychiatric disorders such as schizophrenia – conditions that we considered incompatible with normal integration into the work-force.

### Main exposure

#### Parents’ mental health

The main exposure variables were self-report items from the baseline survey. Adolescents were asked if their parents/caregivers had suffered from mental health problems during the 12 months prior to the survey. The participants were asked to grade the burden of problems into “no, never”, “yes, sometimes”, “yes, many times” and “frequently”. In the descriptive subgroup analyses, we dichotomized the variable into “yes” or “no”.

#### Social support

Social support was measured by the students’ perception of their relationship to their family, friends, classmates and teachers. The answers were given in a 4-point Likert scale. The scales used in the baseline questionnaires were adapted from a paper by Ystgaard and collegues from 1999 [41]. Ystgaard developed the questions for adolescents in accordance with corresponding studies on adults and aimed at measuring availability of support, attachment and mutual care [46].

#### Family support

Family support included five items: “when you think about your family, would you say: I feel attached to my family; my family takes me seriously; my family values my opinions; I mean a lot to my family; I can count on my family when I need help”. The response format was on a scale of 1 (strongly agree) to 4 (strongly disagree). Mean scores were calculated for each scale of five items from respondents who answered at least two items. Mean scores were reversed so that a high score indicated strong perceived support. Cronbach’s alpha for this scale was 0.87.

#### Friends’ support

Friends’ support included four items: “when you think about your friends, would you say: I feel attached to my friends; my friends value my opinions; I can help/support my friends; I can count on my friends when I need help” with responses on a scale of 1 (strongly agree) to 4 (strongly disagree). Mean scores were calculated for each scale of four items from respondents who answered at least two items. Mean scores were reversed so that a high score indicated strong perceived support. Cronbach’s alpha for this scale was 0.83.

#### Classmates’ support

Classmates’ support included four items: “I enjoy my classmates; I have much in common with my classmates; I feel attached to my classmates; and my classmates value my opinions” with responses on a scale of 1 (strongly agree) to 4 (strongly disagree). Mean scores were calculated for each scale of four items from respondents who answered at least two items. Mean scores were reversed so that a high score indicated strong perceived support. Cronbach’s alpha for this scale was 0.81.

#### Teacher support

Teacher support included four items: “my teachers appreciate my opinions; my teachers appreciate me; my teachers help me with my subjects when I need it; and my teachers help me with my personal problems if needed” with responses on a scale of 1 (strongly agree) to 4 (strongly disagree). Mean scores were calculated for each scale of four items from respondents who answered at least two items. Mean scores were reversed so that a high score indicated strong perceived support. Cronbach’s alpha for this scale was 0.82.

### Background covariates

#### Health measures

Self-rated health can predict later morbidity, mortality, use of health services and early disability [47]. In the baseline survey, self-rated health was categorized into four options: “bad”, “not that good”, “good” or “very good”. Mental health problems was scored using the Hopkins Symptom Checklist-10 (HSCL-10), a short-form of the Hopkins Symptom Checklist-25 (HSCL-25), and an instrument designed to diagnose depression *and anxiety* in primary health care [48]. The HSCL-10 includes 10 items about psychological symptoms experienced over the previous week and is validated for use in both general practice and epidemiological studies as a measure on level of internalizing mental health issues [49]. Responses are encoded on a four-point Likert scale from “not troubled” to “heavily troubled”. Mean scores were calculated for each scale of 10 items (range 1–4). Records with three or more missing items were excluded from the analyses.

#### Socio-demographics

The socio-demographic background variables concerning parents’ marital status and household income were based on self-report from the baseline survey. Adolescents were asked whether their parents were “married/living together”, “a single parent”, “divorced/separated”, “one or both dead” or “other”. The question concerning household income was categorized into “very good”, “good”, “mediocre” or “poor”. Information on parents’ educational level was provided by Statistics Norway. The highest completed educational level of one of the parents was used, providing four categories: “higher college or university degree” (>4 years), “lower college or university degree”, “high school” and “primary school”.

### Statistical analyses

For descriptive analyses, we did frequency analyses, together with simple analyses, including Pearson’s Chi-squared tests for differences in general health and socio-demographic factors in the exposed individuals compared with the unexposed. *Cramer’s V was used to evaluate effect size, where the criteria for a small effect =0.01, medium = 0.3 and large = 0.50 in a 2 by 2 table (for test of gender differences in report of mental health problems). In the tables where we had three categories in the row variable, the criteria for a small effect =0.07, medium = 0.21 and large = 0.35* [50]*.* We also tested for gender differences in the outcome variable, using *time to event/Cox proportional hazard* regression analyses. Independent-samples t-tests were used to compare perceived social support measures and the mental health measure (HSCL-10 score) between the exposed and the unexposed groups. *Cohen’s d was used to evaluate effect size, where the criteria for a small effect =0.2, medium = 0.5 and large = 0.8* [50]*.*

To set up survival analyses, we used multiple imputation to account for missing values on the independent variables. We used time to event/Cox proportional hazard regression analysis to calculate hazard ratios (HRs) for time to receipt of the first event of receipt of a long-term welfare benefit, by parents’ mental health problems and social support measures. *The Cox proportional regression model is based on the assumption of proportional hazards, i.e. that the hazard ratio is constant over time. The proportional hazard assumption was checked by Schoenfeld residuals* [51]*. The hazard ratio can be interpreted as a relative instantaneous risk.* We first performed crude analyses on each of the exposure variables separately. In model 1, each exposure was adjusted for socio-demographic background variables; in model 2, we adjusted for adolescents’ health status (general health and mental health) as well. In model 3, we included all background variables described above. In addition, the main exposures, i.e. parents’ mental health and social support, were adjusted for each other. Because of small sample sizes in the groups reporting mental health problems in parents in preliminary gender-stratified analyses, we chose to present unstratified results. Instead, in models 1–3, we adjusted for gender.

Finally, we checked for interactions between parents’ mental health problems and each social support dimension independently. Possible independent interactions between parents’ mental health problems and gender were also assessed, as well as interactions in the fully adjusted model. Because of known possible problems with interaction analyses on multiply imputed data [51], these investigations were also performed on complete cases.

Descriptive analyses of the data were performed using IBM SPSS Statistics version 20.0, while time to event analyses were performed using the R package rms for regression analyses (R Foundation for Statistical Computing, Vienna) and Hmisc (function aregImpute) for generating multiply imputed data.

### Ethical approval

The study was approved by the Norwegian Institute of Public Health, Statistics Norway, the National Insurance Services, the Tax Inspectorate, the Ministry of Education and Research, and the Regional Committee for Medical and Health research Ethics. These institutions gave permission for the use and linkage of the data. The adolescent participants initially gave informed consent to link their survey data to various national registries at follow-up.

## Results

### Main results

After excluding individuals granted a permanent disability pension before the age of 20 and those with missing outcome values, the remaining study sample was 13 976 adolescents. Of these, 49.9% were boys. Sample descriptive data are presented in Table 1. Missing data caused by skipped independent variable items at baseline varied from 0.8% to 6.5%. Of the total sample, 10.3% (1397) reported having parents who suffered from some level of mental health problems during the 12 months prior to the baseline survey; 3.0% (420) reported frequent problems. Significantly more girls reported such experiences (girls 13.6%, boys 7.0%, df = 1, chi-square = 160.0, Cramer’s V = 0.11, P < 0.001).

**Table 1:** **Descriptive characteristics of the total study sample (n = 13 976), those unexposed to a parent with mental health problems and those exposed to such problems (n = 1397) at baseline, 2000–2004**

	**Total population % (n)**	**Unexposed group % (n)**	**Exposed group % (n)**
**Gender**			
*Girls*	50.1 (7004)	48.3 (5873)	66.2 (925)
*Boys*	49.9 (6972)	51.7 (6275)	33.8 (472)
**Social support**	**Mean (SD)**	**Mean (SD)**	**Mean (SD)**
*Family support*	3.59 (0.54)	3.64 (0.50)	3.23 (0.74)
*Friends’ support*	3.60 (0.49)	3.61 (0.47)	3.53 (0.56)
*Classmates’ support*	3.08 (0.69)	3.11 (0.67)	2.84 (0.79)
*Teacher support*	2.91 (0.73)	2.93 (0.72)	2.72 (0.80)
**Self-reported health measures**			
General health	**% (n)**	**% (n)**	**% (n)**
*Very good*	34.0 (4688)	35.6 (4264)	21.9 (302)
*Good*	54.3 (7482)	54.2 (6499)	54.6 (752)
*Not that good*	10.9 (1508)	9.5 (1137)	22.5 (310)
*Bad*	0.7 (100)	0.7 (80)	0.9 (13)
Mental health	Mean (SD)	Mean (SD)	Mean (SD)
*HSCL-10-score*	1.45 (0.49)	1.40 (0.45)	1.85 (0.65)
**Socio-demographic charactristics**	**% (n)**	**% (n)**	**% (n)**
Household income			
*Very good*	9.6 (1313)	9.6 (1157)	7.0 (97)
*Good*	54.0 (7422)	56.1 (6730)	37.5 (517)
*Mediocre*	33.1 (4554)	31.7 (3798)	45.7 (630)
*Poor*	3.3 (459)	2.5 (305)	9.7 (134)
**Parents’ educational level**			
*Highest level of education (>4 years)*	14.0 (1934)	14.2 (1707)	13.1 (183)
*High level of education (≤4 years)*	31.1 (4279)	31.3 (3746)	31.1 (435)
*High school*	41.4 (5697)	41.5 (4974)	41.0 (565)
*Junior high school*	13.5 (1865)	13.0 (1558)	14.8 (204)
**Parents’ marital status**			
*Married or living together*	66.8 (9256)	68.9 (8318)	48.6 (672)
*Divorced/separated*	24.7 (3424)	23.1 (2794)	38.9 (538)
*Single parent*	3.5 (489)	3.4 (413)	4.2 (58)
*One or both dead*	3.0 (421)	2.9 (348)	4.2 (58)
*Other*	2.0 (275)	1.7 (206)	4.1 (57)

Adolescents exposed to parents with mental health problems had significantly worse mean scores on the family *(t = 19.6, df = 1505, P < 0.001, Cohen’s d 0.64)*, friends *(t = 5.1, df = 1612, P < 0.001, Cohen’s d 0.15)*, classmates *(t = 12.3, df = 1616, P < 0.001, Cohen’s d 0.37)* and teacher *(P < 0.001, t = 9.2, df = 1623, Cohen’s d 0.27)* support measures. Those exposed to parents with mental health problems also reported significantly more health problems, including a higher HSCL-10 mean *score (t = 24.6, df = 1498, P < 0.001, Cohen’s d 0.80)* and general health problems (df = 6, chi-square = 278.0, Cramer’s V = 0.10, P < 0.001).

There were no significant differences in the parents’ educational level in the exposed group compared with the unexposed group (df = 6, chi-square = 8.0, Cramer’s V = 0.02, P = 0.25), while the exposed group reported poorer levels of family income (df = 6, chi-square = 411.2, Cramer’s V = 0.12, P < 0.001). In the exposed group, a higher percentage had divorced or single parents (df = 8, chi-square = 265.2, Cramer’s V = 0.10, P < 0.001).

At follow-up, 17.1% (2396) had received some type of long-term welfare benefit. Boys had a slightly higher hazard ratio (HR) for receiving a long-term benefit (girls 15.8%, boys 18.5%, HR 1.16, CI 1.06–1.27, P < 0.001). In the exposed group, 22.5% of those who reported a moderate level of mental health problems in their parents received benefits during follow-up, while 27% of those who reported problems on several occasions received benefits, and 30% of those who reported frequent trouble received benefits (Table 2). Table 2 also shows the proportion of recipients of long-term welfare benefits within gender and background socio-demographic variables, across the total sample.

**Table 2:** **Baseline variables and proportion of use of long-term welfare benefits during the follow-up period in the total sample (n = 13 976)**

	**Long-term benefit**
	**% (n)**
**Parents’ mental health**	
Mental health problems experienced in the past 12 months	
*No, never*	15.8 (1917)
*Yes, sometimes*	22.5 (220)
*Yes, many times*	27.0 (72)
*Frequently*	30.1 (46)
**Gender**	
*Girls*	15.8 (1104)
*Boys*	18.5 (1292)
**Socio-demographic variables**	
Household income	
*Very good*	16.6 (218)
*Good*	14.3 (1059)
*Mediocre*	19.7 (896)
*Poor*	32.5 (149)
Parents’ educational level	
*Higher college or university degree (>4 years)*	5.8 (112)
*Lower college or university degree (≤4 years)*	11.0 (471)
*High school*	19.3 (1097)
*Primary school*	34.3 (640)
Parents’ marital status	
*Married/living together*	13.8 (1276)
*Divorced/separated*	22.0 (752)
*Single parent*	26.6 (130)
*One or both dead*	22.3 (94)
*Other*	34.5 (95)

In the crude Cox regression analyses, we found that adolescent report of mental health problems in parents was associated with long-term receipt of welfare benefits during follow-up, with HRs of 1.49 (CI 1.29–1.71), 1.82 (1.44–2.31) and 2.13 (CI 1.59–2.85) for some trouble, moderate trouble and frequent trouble, respectively, compared with no trouble. Family, friends, classmates and teacher support each analysed separately were all associated with a lower risk of welfare dependence in the crude analyses (Table 3).

**Table 3:** **Associations between measure of exposure to parental mental health problems in 15–16-year-olds (n = 13 976) and later use of long-term welfare benefits through 2010, investigated using Cox regression analysis**

	**Outcome variable**			
	**Crude**	**Model 1**	**Model 2**	**Model 3**
	**Receipt of long-term benefit**	**Receipt of long-term benefit**	**Receipt of long-term benefit**	**Receipt of long-term benefit**
	**HR (95% CI)**	**HR (95% CI)**	**HR (95% CI)**	**HR (95% CI)**
**Parents’ mental health**				
*No, never*	Ref	Ref	Ref	Ref
*Yes, sometimes*	1.49 (1.29–1.71)**	1.34 (1.16–1.54)**	1.19 (1.03–1.37)*	1.15 (0.99–1.33)
*Yes, many times*	1.82 (1.44–2.31)**	1.57 (1.23–2.00)**	1.29 (1.01–1.66)*	1.21 (0.95–1.55)
*Frequently*	2.13 (1.59–2.85)**	1.66 (1.23–2.23)**	1.28 (0.95–1.74)	1.14 (0.84–1.55)
**Family support**	0.76 (0.73–0.79)**	0.83 (0.79–0.86)**	0.88 (0.84–0.91)**	0.84 (0.78–0.90)**
**Friends’ support**	0.83 (0.78–0.88)**	0.91 (0.85–0.96)*	0.96 (0.91–1.02)	1.07 (0.98–1.17)
**Classmate’s support**	0.68 (0.64–0.72)**	0.73 (0.69–0.77)**	0.79 (0.74–0.84)**	0.80 (0.75–0.85)**
**Teacher support**	0.77 (0.73–0.81)**	0.83 (0.79–0.88)**	0.90 (0.85–0.95)**	1.01 (0.95–1.08)

*P <0.05, **P <0.001

Crude: Each main exposure variable tested independently.

Model 1: Adjusted for family economy, parents’ educational level, parents’ marital status and gender.

Model 2: As in model 1, in addition each variable adjusted for the adolescents’ own health status (general health and mental health).

Model 3: As in model 2, in addition parents’ mental health probolems and social support adjusted for each other.

Associations expressed in hazard ratios (HR) with 95% confidence intervals (CI).

In model 1, we adjusted for parents’ educational level, family economy and parents’ marital status. Mental health problems in parents still predicted a higher level of welfare dependence in young adulthood in adjusted analyses, while social support predicted improved outcome. When adjusting for socio-demographic background factors and health measures, in model 2, the associations between some and moderate mental health problems in parents and welfare dependence remained significant, although weaker. Family, classmates and teacher support remained associated with a lower level of welfare dependence. After including all the covariates in one model (model 3), the associations between mental health problems in parents and later welfare dependence were no longer significant (df = 22 in the full model). Support from family and classmates remained a protective factor for welfare dependence in all models, including after correction for all other study covariates (HR 0.83, CI 0.77–0.90 and 0.84, 0.78–0.90).

We investigated the hypothesis that there is a buffering effect of each of the four dimensions of social support by testing for interactions between mental health problems in parents and perceived social support, but found no evidence supporting that the associations between the main exposure, mental health problems in parents, and the outcome, changed within different levels of social support (total P = 0.49, on imputed data total P = 0.67). Nor did we find significant interactions between gender and mental health problems in parents (P = 0.19).

### Testing the proportional hazard assumption

When testing the proportional hazard assumption, we found global deviations (P < 0.001) and significant deviations for some of the predictors in the univariate model, in model 1 and model 3. For classmate support, there was a stronger positive effect in the beginning of the follow-up period, in all the above-mentioned models, while for teacher support the results were more complex and with no consistent interpretation. Teacher support seemed to be a risk factor for an adverse outcome in the beginning of the follow-up period, while the results indicated a protective effect later. In the univariate model, adolescents reporting being frequently troubled by mental health problems in their parents had a higher risk of welfare dependence during the complete follow-up period, though the effect seemed to be stronger in the beginning.

## Discussion

### Main findings

Adolescents who reported mental health problems in their parents at age 15–16, had a higher risk of long-term dependence on welfare assistance during young adulthood compared with their peers who did not report such problems. These associations remained significant after adjusting for socio-demographic factors. After adjusting for the adolescents’ own health status, the associations were attenuated and were no longer significant for frequent problems. After combined adjustment for all study covariates, including perceived social support, associations between parents’ mental health problems and welfare dependence were no longer significant. On the other hand, perceived family and classmate support predicted a significantly improved outcome in all models.

We did not find evidence to support the buffering theory of social support, i.e., that a high level of perceived social support could have a protective effect against adverse outcomes in already burdened individuals.

### Strengths and weaknesses

A major strength of this prospective community study is the substantial number of participants across a geographically diverse area, along with a high response rate and few missing data. The baseline surveys were conducted in six Norwegian counties and encompassed the entire youth population in those regions. The counties located in the southern and northern parts of Norway, included both urban and rural areas and should be representative for a general youth population in Norway. However, not all of those invited participated in the baseline study. Neither did all participants authorize linkage of their survey data to national registry data. Our study is thus based on 76% of the invited 10th graders. Self-selection may bias our results, as the adolescents who refused to participate at baseline or by other reasons did not fill in the questionnaires, possibly could be more disadvantaged or burdened by health problems, compared to those who participated. A selective loss to follow-up may be a limitation to the study. However, empirical evidence supports that generalization associations are less sensitive to loss to follow-up than prevalence measures [52]. In a study based on portions of the same sample as ours, response rates and selection problems were investigated and similar association measures among actual participants and all the invited adolescents were found [52], a fact that supports the main findings of our study.

The prospective longitudinal design of our study is a major strength because it provided the opportunity to follow a large number of individuals over several years, from mid-adolescence to young adulthood. However, given the observational nature of this study, it is important not to draw causal conclusions.

The main exposure – mental health problems in parents – is based on report by the adolescents themselves. We included no objective measure for parent diagnosis; thus, our main exposure measure may be somewhat imprecise. This may have affected the associations with the outcome. Adolescent report may mean an underestimation of mental illness in parents, as well as a lack of detail about type and severity of the problems. Adolescents were asked to report the frequency of the problem during a limited period of time, which is an inaccurate estimate of mental health problems. On the other hand, adolescent report of such problems reflects their own experience, which can be argued to be a reliable measurement of how strongly mental health problems in their parents impact the participant. In other words, if adolescents report that their parents frequently suffer from mental health problems, it is likely that the adolescents also are affected.

Our outcome variable has some limitations. Although the compulsory Norwegian registries provided us with complete, reliable follow-up material, and the quality of the registrations is in general good, errors can occur. Our choice was to define those individuals who were registered on a long-term welfare benefit, according to the previously described definitions, as having problems with work integration. Those who are not qualified for a benefit or who are supported by their family will not be found in the registry datas. Thus, our outcome measure does not necessarily capture all individuals who have trouble with work integration. However, our rates of welfare dependence correspond to findings in other relevant Norwegian studies [30,34,53].

### Comparison with previous research

Several relevant studies from New Zealand [22,54,55] have found associations between mental illness during adolescence and lower educational attainment, lower work participation and increased use of welfare benefits, while few other studies have assessed longitudinal outcomes in children of parents with mental illness. A recent Norwegian study found that adolescents from families in which parents suffered from symptoms of anxiety and depression were at risk of medical welfare dependence in young adulthood, as well as an increased risk of suffering from anxiety and depression themselves during adolescence [34], which is in line with the results of our study.

We found independent, positive associations between all the examined dimensions of social support and later welfare dependence in young adulthood. This is in line with previous research on perceived social support [37] showing a strong relationship with reduced stress and psychological distress, as well as improved well-being in longitudinal studies [41]. A considerable number of studies have investigated the concept of social support and its interaction with stress, coping and emotional and physical well-being; these are outlined in a 2011 review by López and Cooper [35]. Perceived social support, the dimension most frequently studied, refers to an individual’s cognitive appraisal of support to promote coping and thereby reduce the negative effect of stress on outcomes [35]. Family support and positive classmate relationships may strengthen self-esteem and contribute to school connectedness, which in turn may improve general coping and school achievements. However, our results did not support the hypothesized buffering effect in individuals living with parents who have mental health problems.

### Interpretation of findings

Having parents with mental illness is a well-known risk factor for psychological problems and mental health problems in children and adolescents [5,56,57]. One possible explanation by which mental illness in parents can influence future coping and work exclusion in their offspring may be the increased genetic risk of mental illness in the children, which in turn can be a direct cause of work impairment in young adulthood. Parents’ mental health problems are also a stressor in the home environment and can thereby affect children’s health and development. Mental stress can also affect adolescents’ neuropsychological development. Educational attainment and thus the ability to integrate in the labour market in adulthood may thereby be attenuated. Another possible explanation is that other adverse life circumstances that co-exist with mental illness may lead to an increased use of welfare benefits in young adulthood. However, we found that the associations with welfare dependence remained significant after adjusting for well-known confounders such as parent’s educational level, family income, and parent’s marital status, indicating that the effect of parents’ mental problems on the study outcome is partly independent of such factors. The fact that adjustment for health attenuated the associations may indicate that a substantial part of the effect on welfare dependence is explained by ill health in the adolescents themselves.

When also adjusting for social support, the associations between mental health problems in parents and welfare dependence were no longer significant. We consider this finding interesting, as growing up in families with parents who suffer from mental health problems may imply a lower level of perceived family support.

## Conclusions

In Norway 260 000 (23.1%) children live in homes where at least one parent has a mental illness, which can jeopardize daily function [58]. In addition, 115 000 (10.4%) have parents who suffer serious problems [58]. Although many of these children manage well in life, they have an increased risk of experiencing adverse life events, including violence and abuse, failure of care and developing mental illnesses themselves [58]. According to the Norwegian Health Personnel Act [59], health workers in Norway are obliged to provide necessary information and help to under-aged children of parents suffering from serious chronic somatic or mental illness and drug or alcohol addiction. The fact that our study indicates long-term effects of the disadvantage of living with parents with mental health problems calls for a wider perspective when dealing with under-aged children in burdened families. General practitioners, school health service providers and mental health care workers, are all in a good position to identify children with special needs in this context and should offer help and follow-up. Co-operation with the burdened family by strengthening support and coping may help the child. That all the dimensions of social support independently show strong positive associations with lower use of long-term welfare benefits supports a call for a broad approach when caring for both exposed and unexposed children. Building a supportive environment can be of major importance.
